# The impact of persistent organic pollutants on fertility: exposure to the environmental toxicant 2,3,7,8-tetrachlorodibenzo-p-dioxin alters reproductive tract immune responses

**DOI:** 10.3389/fimmu.2024.1497405

**Published:** 2024-12-10

**Authors:** Victoria R. Stephens, Kensley B. Horner, Walter M. Avila, Sabrina K. Spicer, Riya Chinni, Emily B. Bernabe, Antentor O. Hinton, Steven M. Damo, Alison J. Eastman, Monique M. McCallister, Kevin G. Osteen, Jennifer A. Gaddy

**Affiliations:** ^1^ Department of Pathology, Microbiology, and Immunology, Vanderbilt University Medical Center, Nashville, TN, United States; ^2^ Department of Life and Physical Sciences, Fisk University, Nashville, TN, United States; ^3^ Department of Chemistry, Vanderbilt University, Nashville, TN, United States; ^4^ Department of Medicine, Health, and Society, Vanderbilt University, Nashville, TN, United States; ^5^ Tennessee Valley Health Systems, Department of Veterans Affairs, Nashville, TN, United States; ^6^ Department of Molecular Physiology and Biophysics, Vanderbilt University, Nashville, TN, United States; ^7^ Department of Biochemistry, Vanderbilt University, Nashville, TN, United States; ^8^ Center for Structural Biology, Vanderbilt University, Nashville, TN, United States; ^9^ Department of Obstetrics and Gynecology, Vanderbilt University Medical Center, Nashville, TN, United States; ^10^ Department of Biological Sciences, Tennessee State University, Nashville, TN, United States; ^11^ Department of Obstetrics and Gynecology, Meharry Medical College, Nashville, TN, United States; ^12^ Department of Medicine, Division of Infectious Diseases, Vanderbilt University Medical Center, Nashville, TN, United States

**Keywords:** environmental toxicant, TCDD, dioxin, innate immunity, reproduction

## Abstract

Exposure to environmental contaminants can result in profound effects on the host immune system. One class of environmental toxicants, known as dioxins, are persistent environmental contaminants termed “forever chemicals”. The archetype toxicant from this group of chemicals is 2, 3, 7, 8-tetrachlorodibenzo-p-dioxin (TCDD), an immunotoxicant that activates the aryl-hydrocarbon receptor pathway leading to a variety of changes in immune cell responses. Immune cell functions are crucial to the development and maintenance of healthy reproduction. Immune cells facilitate tolerance between at the maternal-fetal interface between the parent and the semi-allogenic fetus and help defend the gravid reproductive tract from infectious assault. Epidemiological studies reveal that exposure to environmental contaminants (such as TCDD) are linked to adverse reproductive health outcomes including endometriosis, placental inflammation, and preterm birth. However, little is known about the molecular mechanisms that underpin how environmental toxicant exposures impact immune functions at the maternal-fetal interface or within the reproductive tract in general. This review presents the most recent published work that studies interactions between dioxin or TCDD exposure, the host immune system, and reproduction.

## Introduction

### Environmental toxicants and forever chemicals

Environmental toxicants are ubiquitous, and chronic exposure is inevitable across the lifespan of most mammals. These chemicals are introduced into the environment as byproducts of natural events (i.e., volcanic eruptions and forest fires), but more commonly through manufacturing and disposal processes (i.e., burning of medical waste and organochloride production) (1). Reported production of manmade chemicals began in the early- to mid-1900s when manufacturers saw their benefits as plasticizers, pesticides/insecticides, preservatives, antimicrobial compounds, pharmaceutical agents, and more (2). By the end of the 20^th^ century, studies began to reveal positive correlations between toxicant exposures and adverse health effects (2). Unfortunately, only a minute proportion of toxicants have been assessed for their toxicity (3), and several well-studied environmental toxicants have been identified as endocrine disrupting chemicals (EDCs) which has led to the reduction and eventual ban of certain compounds (4–6).

The United States Environmental Protection Agency (EPA) previously defined an EDC as “an exogenous agent that interferes with synthesis, secretion, transport, metabolism, binding action, or elimination of natural blood-borne hormones that are present in the body and are responsible for homeostasis, reproduction, and developmental process[es]” (7). Not surprisingly, EDCs have been repeatedly highlighted as a threat to human health. These chemicals can be found in food, beverages, personal care items, plastic children’s toys, cosmetics, as well as many other consumer products (8). The most common routes of human EDC exposures include dietary consumption, dermal absorption, and inhalation. Exposure to this group of chemicals can also occur through the transplacental transfer from mother to fetus, and by lactation from mother to infant (7, 8).

Persistent organic pollutants (POPs) and non-persistent EDCs (npEDCs) are two groups of EDCs. npEDCs have a low lipid solubility hence their relatively short half-life. The body burden of npEDCs is typically reduced as these chemicals are excreted from the body in urine or metabolized to non-bioactive forms (9). On the other hand, POPs, often referred to as “forever chemicals”, are highly lipophilic chemicals that are not readily biodegradable (10). The lipophilicity of many EDCs allows them to penetrate the lipid bilayer of plasma membranes leading to bioaccumulation in adipose tissues. This, in turn, enhances their ability to bio-magnify within the food chain; thus, the primary source of human and animal EDC exposure is the ingestion of contaminated foods (10).

It has been estimated that more than 24% of human disorders and diseases are induced by environmental factors, and 80% of the deadliest diseases (i.e. respiratory disease, cardiovascular diseases, and cancers) are said to have an environmental contribution in their pathology (11). Most of these illnesses are associated with perturbations of the endocrine system which implicates EDCs as a contributing environmental factor (11). Despite increased evidence that links EDCs to adverse effects on human health, a consensus on the cause-and-effect relationship has yet to be reached. However, according to the Endocrine Society, the increasing rate of diseases with endocrine perturbations is occurring simultaneously with the increasing rate of the production of manufactured chemicals (11). For example, the production of plastics has grown from approximately 50 million tons in the mid-1970s to almost 300 million tons today. This trend is consistent for other chemical sources including pesticides and solvents. Indeed, global sales in the chemical industry have increased from 171 billion United States dollars (USD) in 1970 to 4 trillion USD in 2013. In parallel, the prevalence of endocrine-associated disorders and disabilities has increased rapidly as well (11, 12).

### 2,3,7,8-Tetrachlorodibenzo-p-*dioxin*

2,3,7,8-Tetrachlorodibenzo-p-dioxin (TCDD) is an environmental contaminant that belongs to a group of polychlorinated dibenzo-p-dioxins (PCDD), and it is considered to be the prototype and most potent of the dioxin/dioxin-like family (13). TCDD mediates it effects through the aryl hydrocarbon receptor (AHR) whose activation regulates the transcription of numerous genes including those encoding xenobiotic-metabolizing enzymes, known as the AHR battery (14). The AHR battery provides a defense mechanism against potentially toxic environmental contaminants; however, TCDD is resistant to the enzymes which fosters its prolonged occupancy of the AHR and contributes to its potency (15). TCDD is also suggested to be the most biologically active congener of the halogenated aromatic hydrocarbons (HAHs) likely due to being poorly metabolized, hydrophobic, and having a strong affinity for the AHR (13–16). TCDD is chemically stable, and highly resistant to biological and chemical degradation, and thus exhibit a significant degree of bioaccumulation and environmental persistence (17).

TCDD is largely introduced into the environment as an unintentional byproduct of industrial processes. The lipophilicity of TCDD allows it to concentrate in dietary fats such as meat, dairy, fish, and shellfish hence the major source of TCDD exposure to humans is through dietary consumption of contaminated foods (18). The half-life of TCDD is greater than 7 years in humans with respect to timing of exposure, duration of exposure, dose, age, health status, and diet (15). Due to recognized toxicities, policies and regulations have reduced the production and use of several EDC-contaminated and -producing goods; however, TCDD can still be detected in environmental media worldwide (19). Industrialized countries have adopted stricter emission standards and reduced pollution which has led to a drastic decline in TCDD exposure in more recent years (19). In contrast, developing countries are still at risk for high exposure to TCDD due to the incomplete burning of trash, burning of electronic waste and indoor cooking habits (20). Importantly, normal weather patterns can transport airborne contaminants far from their source, making it difficult for any single nation to completely remove these toxins from their environment (20).

Despite increased evidence that links TCDD to adverse effects on human health, pinpointing the direct contribution of TCDD to human disease remains an enigma. Defining a concrete cause-and-effect relationship between these two factors is challenging since humans are chronically exposed to a mixture of chemicals throughout their lifetime. Furthermore, controlling experimental variables by intentionally exposing humans to TCDD and monitoring their outcomes is highly unethical. Therefore, much of our understanding of the link between TCDD and human disease stems from epidemiology studies, cell-based studies, and translational studies using animal models. Using these experimental models, data has consistently provided evidence that TCDD exposure manipulates cells, organs, and organ systems which can adversely affect human health.

TCDD has been detected in human adipose tissue, maternal breast milk, blood, and urine, and it has also been linked to an array of toxicities (21). Notably, TCDD was also a major contaminant of Agent Orange during the Vietnam War (22). Veterans or active-duty military personnel previously or currently deployed near burn pits may represent a population with both acute and transgenerational exposure risks (23, 24). Epidemiological studies have associated TCDD contamination with a variety of disease outcomes including reproductive failure (25). Specifically, early life exposure to TCDD can play a transgenerational role in perturbing immune system functions that reduce reproductive success and alter host development, immune function, and disease risks (20, 24, 25). In this review, we will focus of the effect of TCDD within the reproductive tract, on immune cells, and how maternal-fetal health outcomes are altered by exposure.

### TCDD signals through the aryl hydrocarbon receptor

TCDD primarily mediates its effects through the ligation and activation of the AHR (26–32). Furthermore, sensitivity to TCDD is differential due to distinct AHR alleles, underscoring AHR as a crucial pathway for TCDD activity (29). The AHR is an evolutionary conserved sensor, and a ligand-activated transcription factor that belongs to a superfamily of transcription factors, the periodic circadian protein (PER)–AHR nuclear translocator (ARNT)–single­minded protein (SIM) superfamily (PAS), that senses both endogenous and exogenous stimuli (29). AHR contributes to biological processes based on transcriptional programming determined by signals in the cellular micromilieu (29). This means that AHR can either maintain or disrupt homeostatic conditions potentially leading to a wide range of pathologies, from inflammatory diseases to degenerative diseases, based on ligand specificity (29). Unfortunately, TCDD, as a potent AHR agonist, takes advantage of the power of AHR by binding to it with high affinity to potentially induce maladaptive changes downstream of AHR activation (29).

When inactive, AHR maintains a cytoplasmic localization mediated by a macromolecular complex consisting of 4 proteins: 90 kDA heat shock protein (HSP90), AHR-interacting protein (AIP; commonly referred to as XAP2), p23, and SRC (29). This protein complex also prevents degradation and ensures that AHR maintains a suitable conformation for its ligands. HSP90 binds both the PAS region and the basic helix-loop-helix region of AHR. AIP interacts with the HSP90 dimer and contributes to the proper folding of AHR which inhibits its ability to interact with importin-b, a nuclear import protein (29). Without the co-chaperone protein p23, AHR would be prone to proteasomal degradation mediated by ubiquitylation (29). Lastly, protein kinase SRC contributes to the initiation of AHR activation during its release from the translocation inhibitory complex by triggering a cascade of phosphorylation events on target proteins (29).

Ligand-dependent activation induces the release of AHR from the translocation inhibitory complex, revealing AHR’s nuclear localization signal (NLS) and nuclear export signal (NES) (15). These signals promote AHR translocation to the nucleus, where it forms a heterodimeric complex with the aryl hydrocarbon receptor nuclear transporter (ARNT) (15). This complex functions as a transcription factor, binding to the consensus motif 5′-GCGTG-3′ within the xenobiotic- or dioxin response element (XRE or DRE) in the promoter regions of various genes (15). Once bound, the AHR-ARNT complex engages modulatory proteins that influence transcriptional activity and chromatin conformation resulting in enhanced expression or repression of target genes (15). Interestingly, 65% of TCDD-responsive genes do not inhibit XRE sequences in promoters, indicating pleiotropic and indirect effects, but also that non-consensus XRE elements may confer TCDD inducibility, as demonstrated for PAI-2 and IL-1β (27, 28).

Importantly, AHR-ARNT transcriptional activities are conformation-dependent which is suggested to be directly influenced by ligand binding (30, 31). The AHR modulates various cellular processes, including differentiation, apoptosis, and proliferation, when bound appropriately by endogenous ligands (12). Conversely, inappropriate activation of the AHR by exogenous ligands can have wide-ranging detrimental effects including, but not limited to, the initiation and/or promotion of reproductive failures and disease (12, 29, 33, 34). Additionally, mice lacking AHR have higher numbers of non-apoptotic germ cells compared to wild-type (WT) ovaries cultured in same conditions suggesting AHR contributes to the induction of natural apoptosis in oocytes (35). AHR knock-out mice also have reduced responsiveness of antral follicles to gonadotropins and concomitant reductions in ovulation compared to WT mice. AHRKO females have smaller litters, have difficulty maintaining pregnancy, surviving pregnancy and lactation, and rearing pups to weaning compared to WT females (35).

## TCDD exposure alters cellular functions in the reproductive tract

### The menstrual cycle

Many reproductive failures and gynecologic diseases can be linked to irregularities within a woman’s menstrual cycle. Reproductive health is heavily influenced by the menstrual cycle and its associated hormonal changes (36). The menstrual cycle is defined by the cyclical changes in the uterine lining (endometrium), beginning at puberty and continuing until menopause. While the length of the cycle may vary, the average cycle spans to duration of 28 days. During each menstrual cycle, the endometrium prepares for the potential of implantation and pregnancy. If pregnancy does not occur, the endometrial lining is shed, resulting in menstruation—characterized by the discharge of blood, cells, tissue, and mucus from the vagina (36).

The menstrual cycle is largely governed by a hormonal feedback loop. Once puberty begins, the hypothalamus secretes gonadotropin-releasing hormone (GnRH) (37). GnRH is then transported to the anterior pituitary where it activates the GnRH receptor (37). This activation triggers the secretion of follicle-stimulating hormone (FSH) and luteinizing hormone (LH) by the anterior pituitary which triggers steroid-producing activities in the ovaries (37). LH acts on ovarian theca cells to produce and secrete progesterone and androstenedione (37). Androstenedione is then utilized by ovarian granulosa cells to produce 17-beta-estradiol, utilizing FSH and aromatase (37). Depending on the phase of the menstrual cycle, the anterior pituitary receives signals that regulate the production of FSH and LH which, in turn, influences the production of 17-beta-estradiol and progesterone (37).

The menstrual cycle is divided into three phases: the proliferative phase, the secretory phase, and the menstrual phase (37). The proliferative phase, based on a 28-day cycle, begins on day 5 and ends on day 14 (37). 17-beta-estradiol is the dominant hormone during this phase, and its purpose is to facilitate endometrial proliferation and regrowth. 17-beta-estradiol increases the number and size of the glandular invaginations of the stroma and increases the depths of the spiral arteries for the nourishment of the endometrium (37). Ovulation occurs on day 14 which marks the end of the proliferative phase (37). The secretory phase is the next phase of the cycle, and it begins on day 14 and ends on day 28. Progesterone is the dominant hormone in this phase, and it represses endometrial proliferation while increasing vasculature and mucus secretions to prepare an endometrium suitable for implantation. If implantation does not occur, progesterone levels drop rapidly (37). This hormonal decline triggers the onset of the menstrual phase, marking the beginning of a new cycle (37). The menstrual phase begins on day 1 and ends on day 5 in an average 28-day cycle. The endometrial changes from the previous cycle can no longer be sustained, leading to endometrial sloughing (37). This process expels blood, tissue debris, mucus, and a host of other factors in preparation for the next menstrual cycle (37).

Unfortunately, TCDD has proven to be capable of manipulating various components of the menstrual and ovarian cycles (38). The feedback mechanism that governs the menstrual and ovarian cycles is largely predicated on the regulation of GnRH to control hormone production (37). Studies provide evidence that single doses of TCDD (0.03-30 μg/kg) increases GnRH in a dose-dependent manner which, in turn, increases LH and FSH in female rats (38). Interestingly, another study found that TCDD (ED50 20 μg/kg) inhibits the increase of GnRH receptors and LH secretion in male rats (39). Li et al. specifically assessed the effects of TCDD exposure on ovulation using a gonadotropin-primed immature female rat model (38). Results revealed that TCDD (ED50 3-10 μg/kg) reduced ovulation in exposed animals as indicated by decreased ovarian weight gain and ova recovery from the TCDD-exposed mice (38). They also found that TCDD reduced the peak concentrations of FSH and LH yet increased the concentration of estradiol-17 beta in TCDD-treated mice compared to controls (38). The disparities in these findings may stem from variations in the animal models and the timing of exposure across the studies. In previous studies, TCDD dosing has been tested using the World Health Organization’s consensus dose values for toxic effects (40). Specifically, the investigators utilized dosing levels commonly found in food sources which include a range of 0, 0.05, 0.2, 0.8, or 1.0 μg of TCDD per kg (40). Thus, the dosing used in many of these studies might represent acute high exposure such as those that might be encountered from disaster sites.

As previously mentioned, estrogen and progesterone are the two main hormones of the menstrual cycle (37). Progesterone mediates its actions through its highly specific receptor, the progesterone receptor (PGR) (37). Nayyar et al. revealed that a prenatal TCDD (10 μg/kg) exposure reduced the expression of PGR in the uteri of adult, female mice (41). There are two isoforms of the PGR: PGR-A and PGR-B. PGR-A acts as a repressor of the anti-inflammatory action of progesterone, which is largely mediated by PGR-B. Igarashi and colleagues assessed the impact of TCDD on the PGR ratio of normal human endometrial-stromal cell co-cultures (42). Data revealed that an *in vitro* TCDD (0.1-20 nM) exposure lowered the PGR-B:PGR-A ratio in women with endometriosis (42). Furthermore, they observed an inability of progesterone to down-regulate both stromal-specific pro-matrix metalloproteinase (MMP)-3 and epithelial-specific pro-MMP-7 as a result of the TCDD-induced disruption of stromal PGR expression (43). MMPs are enzymes that mediate normal tissue turnover, including endometrial breakdown at menstruation (43). They are also expressed in association with estrogen-mediated growth, but the role of these enzymes is dictated by the differentiating influence of progesterone (44). If progesterone action is lost, MMPs can continue to foster the breakdown of the endometrium which can lengthen the menstrual phase (44). Furthermore, reduced progesterone activities decrease its ability to counteract the proinflammatory signaling induced by estrogen (44).

TCDD is widely known to mediate its action through the AHR; however, several studies have highlighted its ability to act as an estrogen mimic through ligand binding to the estrogen receptor (ER) (45). Using an estrogen-responsive reporter transgenic mouse harboring a luciferase gene expressed in the presence of estrogenic signaling, Yoshida et al. observed an increase in luciferase activity in the pituitary gland and hypothalamus in mice treated with TCDD compared to vehicle-treated mice (45). This finding was further enhanced in mice treated with TCDD and 17β-estradiol suggesting that there is a synergistic effect on estrogen signaling. Boverhof and colleagues also revealed that many of the transcriptional responses in uterine gene expression induced by TCDD involve the ER (46). Data from TCDD-treated or ethynyl estradiol-treated ovariectomized mice revealed numerous changes in gene expression in response to these compounds. Furthermore, pretreatment with the ER antagonist, ICI 182 780 (faslodex), inhibited gene expression in the uteri of mice in both treatment groups which further validated their findings (46). As it relates to the menstrual cycle, persistent estrogenic signaling, as well as TCDD exposure, induces maladaptive pathophysiological changes to the endometrium and reproductive tract that have been associated with numerous endometrial-related disorders, reproductive failures, and gynecologic diseases (47–49).

## TCDD exposure leads to adverse effects on reproductive health

### TCDD and endometriosis

**Figure 1:**
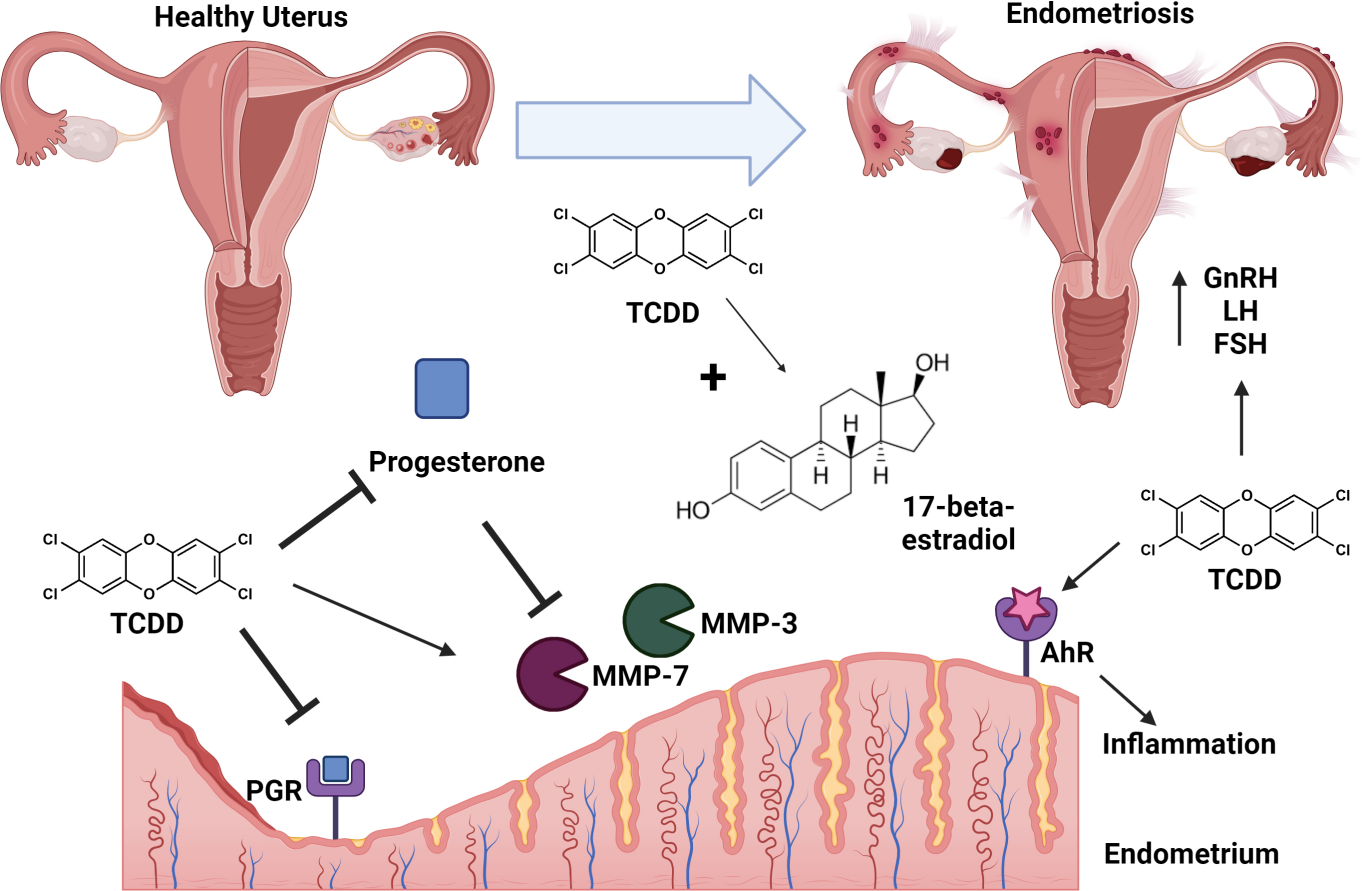
Conceptual model of TCDD exposure and endometriosis. TCDD exposure leads to adverse effects in the uterus including repression of progesterone production and inhibition of progesterone receptor (PGR) signaling. TCDD synergizes with 17-beta-estradiol leading to signaling cascades that enhance risk for development of endometriosis. TCDD-dependent inhibition of progesterone results in de-repression/enhanced activity of matrix metalloproteinses (MMP-3 and MMP-7). TCDD exposure is associated with enhanced levels of gonadotrophin releasing hormone (GnRH), follicle-stimulating hormone (FSH), and luteinizing hormone (LH) which enhance risk of endometriosis. TCDD-dependent signaling through the aryl-hydrocarbon receptor (AhR) results in enhanced inflammation leading to endometriosis. Endometriosis is characterized as lesions of ectopic implantation of functional tissue lining the uterus. Figure created with Biorender.

One notable consequence of TCDD exposure includes the immunological, endocrine, and inflammatory manipulations that contribute to the development of endometriosis (**Figure 1**) (50). Endometriosis is a reproductive disorder characterized by the growth of endometrial tissues at extrauterine sites (50). Typically, when the immune system detects endometrial tissue outside the uterine cavity, it mounts an immune response to eliminate the endometrial debris. In the absence of the necessary immune responses, endometrial tissue remains present, potentially leading to immunological dysregulation and symptoms such as pelvic pain and dysmenorrhea, which are common indicators of endometriosis (50).

Activation of the AHR has demonstrated immunomodulation of inflammatory processes that contribute to the development of endometriosis, specifically through increased expression of AHR genes on peripheral blood mononuclear cells (PBMCs) in the presence of TCDD, particularly in individuals with endometriosis (51, 52). On a molecular level, TCDD may use microRNAs to modulate gene transcription by introducing epigenetic modifications, which can affect AHR-mediated immunomodulatory processes related to cytokine and chemokine production (51). Specifically, IL-10, an interleukin cytokine produced by Th17 cells, is known to promote the development of ectopic lesions characteristic to endometriosis and is highly secreted when AHR is overexpressed (53, 54).

Furthermore, TCDD’s ability to alter the expression of PGR, and specifically favor PGR-A, aids in its ability to contribute to the development of endometriosis. The link between PR-A and endometriosis had been previously established by Attia and colleagues, as PR-A transcripts and proteins are detected in endometriotic tissue samples, while PR-B transcripts and proteins could not be found (55). In contrast, both PR-A and PR-B transcripts and proteins were detected in endometriotic tissue samples obtained from the uterine cavity (55).

Additionally, secretory-phase endometrial-epithelial cells predominantly produce glycodelin (GdA), an immunosuppressive glycoprotein crucial for embryonic implantation and the establishment of pregnancy (56). Interestingly, patients with endometriosis show elevated GdA levels in serum and peritoneal fluid compared to those without disease (57). This elevation, alongside evidence of GdA’s contraceptive effects, suggests that GdA could play a role in endometriosis (58). Specifically, GdA has been shown to inhibit leukocyte functions, preventing their defense against the accumulation of endometrial debris in the peritoneal cavity of cycling women, a key risk factor for endometriosis (59–62).

Considering TCDD’s impact on female reproductive organ development and function, Mueller et al. investigated whether TCDD directly affects GdA production in human endometrium (63). Using endometrial explants (EE) obtained from ovulatory women in the proliferative or secretory phase, Mueller and colleagues cultured EEs in the presence or absence of TCDD. Data revealed a 2.5-fold increase in GdA production in EEs treated with 10 nM TCDD for 9 days compared to untreated EEs (63). Fluorography showed a 3-to 4-fold increase in new GdA biosynthesis and secretion in TCDD-treated endometrial-epithelial cells (63). Moreover, using EEs and cell cultures transfected with luciferase fusion vectors and an AhR expression vector, researchers demonstrated that TCDD increases GdA transcription by binding to dioxin responsive element (DRE) in an AHR-dependent manner (63). The direct AHR-mediated impact of dioxin on GdA gene transcription and protein secretion suggests a potential connection between TCDD exposure and endometriosis, a known risk factor for preterm birth.

### TCDD and preterm birth

Preterm birth is the leading cause of perinatal mortality, accounting for approximately 30% of neonatal deaths (64). PTB is defined as parturition that occurs before the completion of 37 weeks of gestation in humans whereas PTB is defined as spontaneous labor 12-24 hours prior to term (19.5 days after identification of a vaginal plug in C57BL/6 mice) in mice (65). Mammalian parturition is an inflammatory process largely mediated by decreased progesterone activity. As previously mentioned, mice with a history of a prenatal TCDD exposure (F1 mice) exhibit reduced uterine PGR expression; therefore, it is not surprising that studies have also revealed an increased rate of preterm birth (PTB) in these animals (66). In a study conducted by Ding and colleagues, data revealed that 39% (11/28) of F1 female mice mated to control males became pregnant, of which 36% (4/11) delivered preterm (66). Additionally, control females mated with F1 males achieved a pregnancy rate of 47% (18/38), with a 39% preterm birth rate (7/18). In contrast, all unexposed control females mated to control males (15/15) delivered full-term (66). These data suggest that prenatal TCDD exposure in both maternal and paternal lineages increases the risk of preterm birth.

### TCDD and placental inflammation

The placenta is a transient fetal organ that facilitates the exchange of nutrients from mother to fetus, supports fetal growth, and removes waste products from fetal circulation (67). This organ plays a critical role in sustaining both the mother and fetus during pregnancy. Researchers have discovered that paternally expressed genes predominate the organ; thus, the father has a sizable impact on the health and development of the placenta as well as the maintenance of pregnancy (67).

In a previously published study, Ding et al. assessed the impact of an *in utero* TCDD exposure on placental inflammation using F1 mice (66). Data revealed that a developmental toxicant exposure significantly increased premature placental inflammation as indicated by the heightened expression of TLR-4 mRNA in preterm placenta from gestating F1 females mated to control males. They also observed a more significant increase in TLR-4 mRNA expression in preterm placenta from gestating control females mated to F1 males. Additionally, Peltier et al. identified a shift towards inflammation in second-trimester placental explants stimulated with TCDD (68). Specifically, they found an increase in PGE2 and cyclooxygenase (COX)-2 gene expression, increased IL-1β, and decreased IL-10 production in the explants after 72 hours of TCDD stimulation compared to untreated controls (68). Furthermore, the aryl-hydrocarbon receptor has been implicated in TCDD-dependent changes in placental inflammation (69). These data suggest that reduced PGR expression in concert with placental inflammation contributes to the increased risk for preterm birth in toxicant-exposed animals while highlighting the significance of the paternal contribution to the overall development and health of the placenta.

### Multi- and trans-generational fertility issues

TCDD is believed to not only impact the victims of direct exposure, but future generations as well (70). Numerous *in vivo* studies have underscored the potential for the multigenerational and transgenerational impact of parental or ancestral TCDD exposures, which may contribute to the development of reproductive failures in adulthood (20, 66, 70). Bruner-Tran et al. established a mouse model of *in utero* toxicant exposure that allows for a unique opportunity to study multi- and transgenerational reproductive outcomes (20, 66). Fetuses *in utero* and germ cells residing in the F1 fetuses (F1 generation and F2 generation, respectively) are directly exposed – a multigenerational exposure. Effects within the F3 generation and beyond are believed to result from germline epigenetic changes due to ancestral exposures. Numerous endometriosis-like phenotypes were reported in female mice (a phenotype observed in up to four generations) that were markedly similar to women with clinically confirmed endometriosis (20). Using this model, they have also examined the sperm of TCDD-exposed male mice revealing a decrease in sperm number as well as an increased presence of abnormal sperm morphology (71). Testicular inflammation associated with apoptosis of developing spermatocytes was also revealed in these animals. These findings persisted in three generations, but, notably, F2 males and females experienced the most adverse reproductive health compared to the other generations of TCDD-exposed mice using this model (71).

Manikkam et al. observed multi- and transgenerational fertility issues in rats that resulted from a TCDD exposure in gestating females of the founding generation of their model (72). In F1 and F3 females, they identified an increase in polycystic ovary disease, ovarian primordial follicle loss, and pubertal abnormalities (specific to F3 females) whereas F1 males displayed an increase in prostate disease (72). Interestingly, they found that the epigenome of sperm from F3 males contained 50 differentially DNA methylated regions (DMR) in gene promoters (72). Together, these observations demonstrate that TCDD exposure in a gestating female induces multigenerational and transgenerational inheritance of fertility issues in rodents.

In 1976, a chemical plant exploded near Seveso, Italy, and led to the highest known exposure to TCDD in a residential population (73, 74). In 2014, researchers launched the Seveso Second Generation Study which was created to assess the impact of *in utero* TCDD exposure in the offspring of participants of the Seveso Women’s Health Study (SWHS), a retrospective cohort study of women who were directly exposed to TCDD due to the explosion (73, 74). Similar to the women of SWHS, their daughters exhibit decreased fecundability and an increased risk of infertility (73, 74). Importantly, adult males who were born to mothers living in the exposed area have been found to have reduced sperm count, motility, and concentration (73, 74).

Another large-scale TCDD exposure occurred as a direct result of Operation Ranch Hand (75). This U.S. military operation involved the aerial spraying of herbicides to destroy groundcover in Vietnam during American military actions (75). Agent Orange, the herbicide used in this operation, was a 50:50 mixture of 2,4,5-Trichlorophenoxyacetic acid (2,4,5-T) and 2,4-Dichlorophenoxyacetic acid (2,4- D) (76). Unfortunately, TCDD is a contaminant of 2,4,5-T; therefore, Operation Ranch Hand led to unintentional TCDD exposure to troops, residents of the nearby inhabited villages, and those who consumed agricultural products from that area (75, 76). A wide range of health issues have been observed in exposed individuals including reproductive failures such as pregnancy loss and spontaneous PTB (77).

### Ahr is implicated in a variety of immune pathways

The initiation, development, and progression of many reproductive failures and gynecologic conditions are suggested to be a consequence of immune dysfunction. Since AHR is expressed by numerous cells of the immune system, it is likely that EDCs such as TCDD mediate maladaptive downstream effects of misregulated AHR activation (78). AHR is a cellular sensor activated by the ligation of small molecules that enter the cellular micromilieu through the environment, diet, metabolism, microbial flora, and pollutants as well as by small molecules produced in the endogenous milieu (79). AHR integrates these various signals and, in turn, affect the responses of the immune system (29). However, knowledge regarding how environmental factors regulate this vulnerable system remains scarce. Fortunately, AHR signaling provides an investigative mechanism to help decipher the immunomodulatory properties of environmental cues.

Once AHR translocates to the nucleus and binds to the ARNT as previously discussed, there are a variety of ways in which inflammatory pathways can be modulated (29). The AHR-ARNT complex is known to control the transcription of multiple immune-associated genes (29). The core consensus motif 5’-GCGTG-3’, also known as the dioxin responsive element (DRE), embedded within the regulatory region of inflammatory genes that are targets of the AHR (80). This allows for the AHR-ARNT complex to bind to DREs to regulate inflammatory genes involved in the response to dioxin while also interacting with the neighboring transcriptionally regulatory regions of target genes (80). The nucleotide heterogeneity upstream and downstream of the DRE allows for an increased diversity of responses once bound by the AHR-ARNT transcriptional complex (80).

Furman et al., found putative DREs located within the regulatory regions of several key transcription factor genes that are activated during an immune response including several nuclear factor kappa beta (NF-kB) family members such as *NFkB, RELB, and c-REL* (80). NF-kB plays a pivotal role in regulating several inflammatory mechanisms in both the adaptive and innate immune system. This transcription factor induces gene expression of pro- and anti-inflammatory cytokines, chemokines, and adhesion molecules (81). It has also been found to participate in the activation and inhibition of the inflammasome through its activities in regulating nucleotide-binding oligomerization domain, leucine rich repeat and pyrin domain containing protein 3 (NLRP3) (82). Additionally, the development, activation, differentiation, and survival of multiple immune cells depends on NF-kB (83). *IRF1* and *IRF4* are genes involved in the interferon regulatory factor (IRFs) family of transcription factors which, like NF-kB, contributes to immune cell development, differentiation, and their anti-viral and antimicrobial responses (84). They also observed that many genes that encode cytokines and other regulatory factors of an immune response carried DREs within their promoter regions (85). Not surprisingly, misregulated gene transcription caused by inappropriate DRE activation can lead to improper immune responses that potentially result is poor health and disease.

DREs in the majority of the named genes were no more than approximately 200 bp from the transcription start site in the promoter region of the target genes (80). The DREs in a few of the genes also colocalized within the promoter region. It is presumed that the colocalization of the DREs in the promoter regions, especially near the transcriptional start site, increases the reactivity of the target genes to a lower concentration of dioxin (80). DREs also contain enhancer properties increasing their potential to be functionally active. Furthermore, since transcription factors, cytokines, and other inflammatory mediators express DREs-containing genes, xenobiotics such as dioxin can initiate and/or manipulate immune responses through AHR binding activities.

### TCDD impacts the host immune system

Macrophages are phagocytic, scavenger cells that are instrumental in eliciting immune responses (85). Macrophages work by sensing, engulfing, and destroying foreign bodies such as bacteria (85). Their effector functions also require the secretion of antimicrobial mediators including cytokines which stimulate and signal to other cells of the immune system (85). Macrophages not only respond to external challenges, but they are also essential for development and the maintenance of tissue homeostasis especially during physiological changes within the body such as pregnancy (85, 86).

TCDD exposure has been found to significantly alter the production of cytokines in macrophages (87–89). TCDD (up to 40nM) exposure also upregulates the mRNA expression of cytokine-induced, inflammatory marker cyclooxygenase-2 (COX-2) in *E. coli* stimulated and unstimulated placental explants (68). However, the inflammatory responses to infection are severely attenuated. In several infection-related inflammation models, TCDD-exposed host immune cells have been shown to experience significantly blunted production of cytokines and chemokines necessary for a proper immune response (90–92). Wang et al. found that the hyper-expression of RANTES in women with endometriosis induced tolerogenic macrophages (92). Additionally, CCL5/RANTES and CXCL4 have been identified as TCDD-regulated genes in the reproductive system, underscoring how dioxin exposure can dysregulate cytokine responses (93, 94). These findings shed light on the possibility that premature cytokine/chemokine expression, as mentioned above, could have the same effect in relation to infectious outcomes. TCDD-exposed macrophages also exhibit decreased mRNA expression of adhesion molecules ICAM-1, VCAM-1, and CD11b as well as F-actin which contributes to a decrease in cell pseudopodia and adherence (88). Together, TCDD-induced reductions in motility and premature activation negatively regulate macrophage responses to infection.

### TCDD and placental macrophages

**Figure 2:**
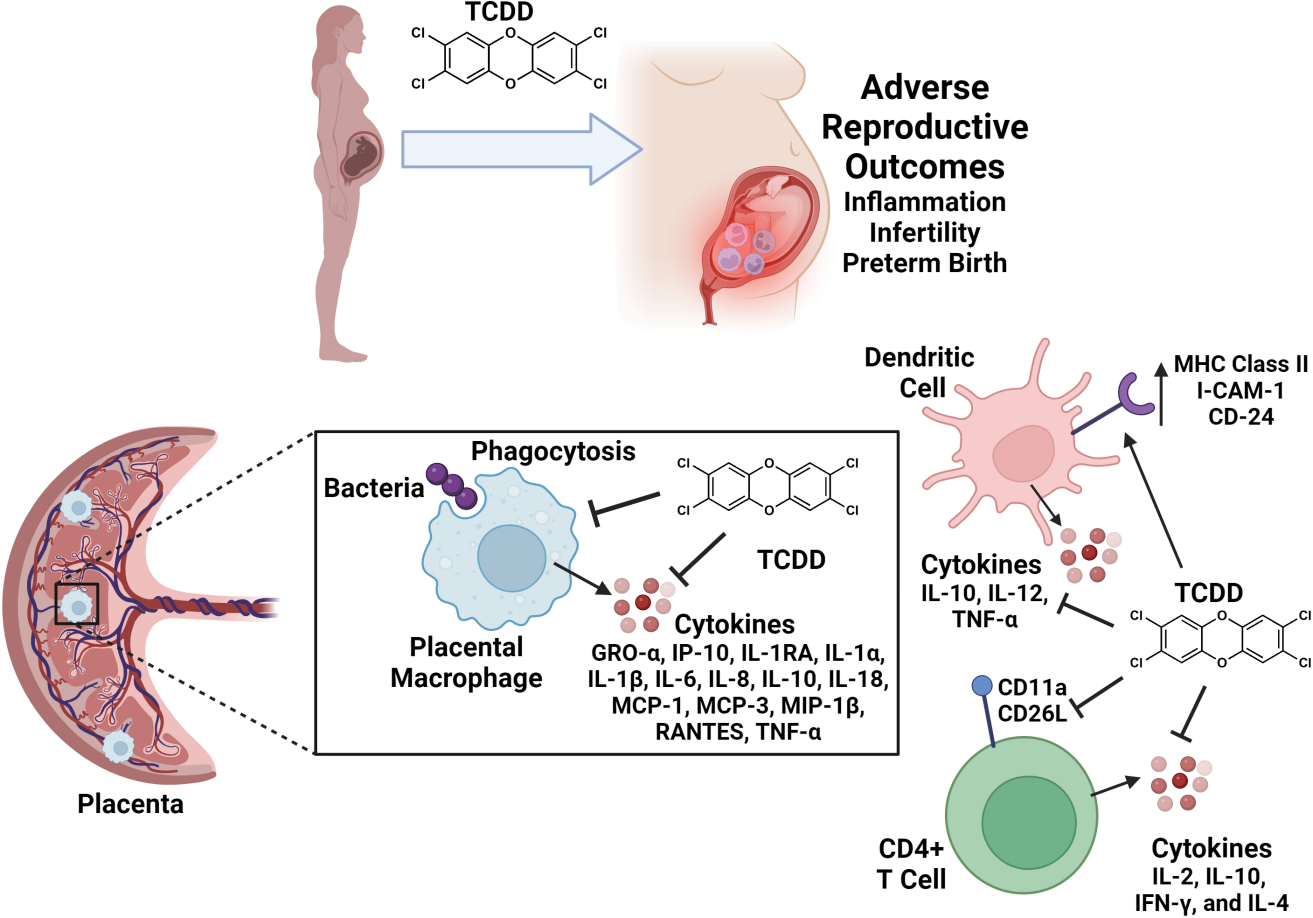
Conceptual model of reproductive immune cell disruption as a consequence of TCDD exposure during pregnancy. TCDD exposure leads to adverse effects on reproduction including inflammation, infertility, and preterm birth. TCDD alters immune cell functions in gravid reproductive tissues and in circulating immune cell populations. TCDD exposure inhibits placental macrophage phagocytosis and cytokine responses to bacterial infection. TCDD enhances dendritic cell surface expression of I-CAM-1, CD-24, and MHC Class II molecules. Dendritic cell exposure to TCDD inhibits IL-10, IL-12 and TNF-α secretion. TCDD exposure inhibits CD11a and CD26L expression and production of IL-2, IL-10, IFN-γ, and IL-4 cytokines in CD4+ T cell populations. Figure created with Biorender.

One of the most crucial immune cell types specific to the gravid reproductive tract are placental macrophages, or Hofbauer cells (95). Placental macrophages exhibit general microbicidal functions, including phagocytosis, production of reactive oxygen species, antigen presentation, and cytokine release (**Figure 2**) (96). These specialized macrophages also participate in the remodeling of tissues at the maternal-fetal interface, as well as inflammatory regulation for semi-allogeneic tolerance (86, 97). Polarization of placental macrophages into differing functional groups demonstrates the plasticity of this cell type, with M1 macrophages being those that engage in microbial killing and inflammation, and M2 macrophages initiating immunosuppressive pathways and tissue repair (98).

A recent study indicates that TCDD exposure impairs placental macrophage activity in the event of invasive bacterial infection (99). In this study, placentas were collected from healthy patients undergoing term, non-laboring c-section delivery, and placental macrophages were isolated to test under varying, physiologically representative TCDD concentrations of exposure (99). Results from their first experiment show that as little as 5 nM TCDD is lethal to placental macrophages. Another study on THP-1 macrophages indicates a lethal concentration of 60 nM TCDD or higher, which comparatively suggests that placental macrophages display a greater sensitivity to TCDD (100). Exposure to sub-lethal concentrations of TCDD similar to those reflective of chronic exposures resulted in attenuated ability of placental macrophages to phagocytize and internalize GBS (99). Lastly, experiments from this study also show that cytokine release was inhibited by TCDD in placental macrophages infected by GBS (99). Taken together, these results indicate that TCDD exposure paralyzes placental macrophage responses to microbial threat (**Figure 2**).

Dendritic cells (DCs) are antigen-presenting cells that serve as a link between the two arms of the immune system – the innate immune system and the adaptive immune system (101). DCs are sentinel innate cells that detect, capture, process, and present antigens to lymphocytes of the adaptive immune system to initiate mechanisms that confer protection against infectious pathogens (101). DCs upregulate their expression of major histocompatibility complex (MHC) class II molecules and migrate to the lymph nodes to present antigens to T cells (102). They also express costimulatory surface ligands and produce cytokines that activate CD4+ T helper cells which, in turn, activate antibody-producing B cells to mount a protection against the pathogen leading to the eradication of infection (102). However, TCDD exposure has been shown to alter the function and presence of DCs and T cells (103–108).

Splenic and bone marrow-derived DCs exposed to TCDD have been shown to have increased expression of MHC class II molecules, adhesion molecules (intercellular adhesion molecule type 1 (ICAM-1) and CD24), and co-stimulatory molecules (B7-2 CD40) in the absence of antigens (102). TCDD-exposed DCs were also shown to exhibit an increased production of interleukin (IL)-12 which regulates the Th1 response by enhancing T cell proliferation and modulating the activities of cytotoxic T lymphocytes (103). While TCDD appears to promote the T cell stimulatory capacities of DCs, the premature activation and stimulation of TCDD-exposed cells significantly reduces the number of DCs which is likely a consequence of an increased rate of Fas-mediated apoptosis (105). Apoptosis of DCs is crucial for regulating the balance between tolerance and immunity (101, 102). However, premature activation and subsequent loss of DCs indirectly contribute to TCDD-induced suppression of the T cell-mediated immune response in the presence of antigens. This occurs by diminishing the strength and duration of the immune response due to inadequate DC stimulation of T cells. Relevant to this notion, Simones and Shepherd reported that TCDD-exposed DCs secreted significantly less IL-6, tumor necrosis factor- (-α), IL-10, and IL-12 in the presence of inflammatory challenges (**Figure 2**) (104). Overall, these studies conclude that premature activation of DCs by TCDD negatively regulates immune responses.

#### T cells

T cells are a diverse group of lymphocytes that mediate many aspects of the adaptive immune system including the response to tumors, allergens, vaccines, pathogens, infections, and environment (105, 108). Once an antigen is bound to a receptor on the surface of the T cell, the T cell undergoes clonal expansion, and, in response to cytokines and other co-stimulatory signals, they differentiate and gain effector functions that aim to eliminate the antigenic stimulus (108). Once the threat is eliminated, antigen-reactive T cells enter a state of quiescence, yet maintain memory, which allows for a more rapid and potent response in the presence of a subsequent, identical, antigenic stimulation (108). Unfortunately, the effector functions of T cells are modulated in the presence of TCDD (108).

CD4+ and CD8+ T cells in TCDD-treated mice have been shown to undergo an initial expansion and proliferate comparable to T cells in untreated mice in the presence of an antigenic challenge; however, these cells disappear much more rapidly in the presence of TCDD (105). TCDD-exposed CD4+ T cells produce significantly less IL-2, IL-10, IFN-γ, and IL-4 which suggests that TCDD modulates CD4+ T cells survival and/or differentiation (105, 107, 108). CD62L has also been shown to be downregulated in CD4+ T cells of TCDD-treated mice highlighting a decrease in T cell activation (105). As previously mentioned, DC-T cell interactions are essential for immunity. Unfortunately, CD4+ T cells in TCDD-treated mice have been shown to have a defect in their ability to up-regulate the adhesion molecule CD11a which decreases its ability to interact with DCs (**Figure 2**) (103, 104). Activation-induced cell death (AICD) is a process by which activated T cells clonally expand then undergo cell-autonomous apoptosis to protect the host from uncontrolled activation, proliferation, and effector functions. Studies have shown that TCDD is capable of increasing apoptosis in CD4+ T cells demonstrating a premature induction in AICD which could explain the rapid decline of antigen-primed T cells in TCDD-treated mice compared to untreated mice (105). These results implicate the repertoire of changes in immune function due to TCDD exposure in dysregulation of healthy pregnancy maintenance and development.

## Conclusions

**Figure 3:**
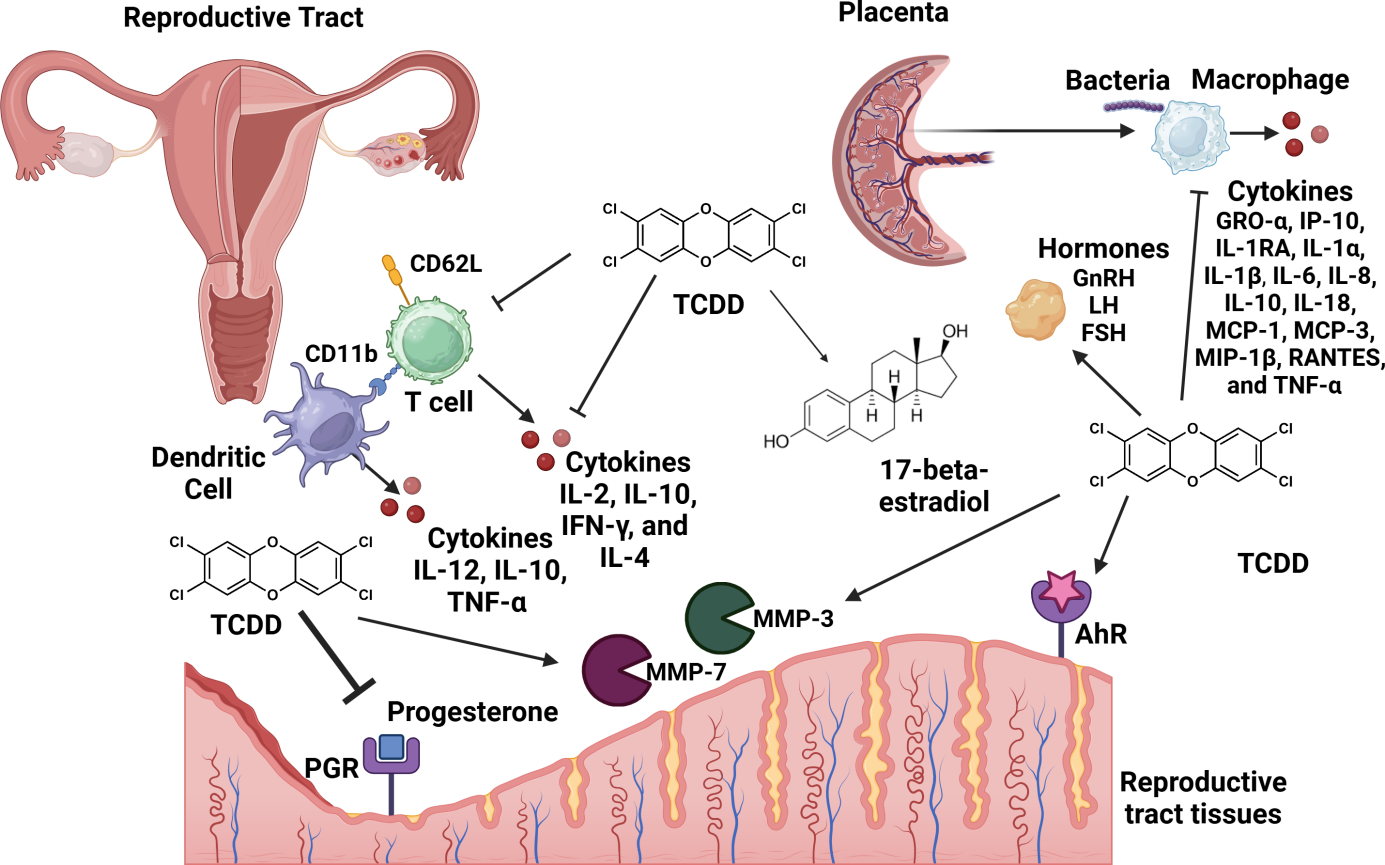
Conceptual model of the consequences of TCDD exposure to the reproductive tract. TCDD exposure leads to adverse effects in the reproductive tract including repression of progesterone production and inhibition of progesterone receptor (PGR) signaling. TCDD enhances the activity of 17-beta-estradiol. TCDD enhances the activity of tissue remodeling matrix metalloproteinases (MMP-3 and MMP-7). TCDD enhances hormones such as: gonadotrophin releasing hormone (GnRH), follicle-stimulating hormome (FSH), and luteinizing hormone (LH). TCDD-dependent signaling through the aryl-hydrocarbon receptor (AhR) results in enhanced inflammation in the reproductive tract. TCDD exposure inhibits placental macrophage phagocytosis of bacterial cells and cytokine responses to infection. Dendritic cell exposure to TCDD inhibits IL-10, IL-12 and TNF-α secretion. TCDD exposure inhibits CD11a and CD26L expression and production of IL-2, IL-10, IFN-γ, and IL-4 cytokines in CD4+ T cell populations. Figure created with Biorender.

In conclusion, exposure to environmental toxicants such as TCDD result in pleiotropic effects that cross tissue types and generations, resulting in a variety of disease outcomes. The reproductive tract is extremely sensitive to dioxin toxicity, which results in perturbations in inflammatory pathways as well as paralysis of innate immune responses in the context of pregnancy (**Figure 3**). Taken together, these results indicate that a better understanding of the molecular mechanisms that underpin dioxin-dependent immunotoxicity of the reproductive tract could yield novel therapeutics or diagnostics to help ameliorate infertility as well as other disease outcomes that occur as a consequence to TCDD exposure.
